# Improving plasma sprayed Raney-type nickel–molybdenum electrodes towards high-performance hydrogen evolution in alkaline medium

**DOI:** 10.1038/s41598-020-67954-y

**Published:** 2020-07-02

**Authors:** Fatemeh Razmjooei, Taikai Liu, Daniela Aguiar Azevedo, Efi Hadjixenophontos, Regine Reissner, Günter Schiller, Syed Asif Ansar, Kaspar Andreas Friedrich

**Affiliations:** 1 Institute of Engineering Thermodynamics, German Aerospace Center, Pfaffenwaldring 38-40, 70569 Stuttgart, Germany; 2 Guangdong Institute of New Materials, Changxing Road No. 363, Guangzhou, 610565 China; 3 Department of Chemical Engineering, Faculdade de Engenharia, Universidade do Porto, 4200-465 Porto, Portugal; 4 Institute of Building Energetics, Thermal Engineering and Energy Storage (IGTE), University of Stuttgart, Pfaffenwaldring 31, 70569 Stuttgart, Germany

**Keywords:** Energy science and technology, Engineering

## Abstract

Rationally designed free-standing and binder-free Raney-type nickel–molybdenum (Ni–Mo) electrodes produced via atmospheric plasma spraying (APS) are developed by correlating APS process parameters with the microstructure of electrodes and their electrochemical performance in alkaline media. The results revealed that the electrode morphology and elemental composition are highly affected by the plasma parameters during the electrode fabrication. It is found that increasing plasma gas flow rate and input plasma power resulted in higher in-flight particle velocities and shorter dwell time, which in result delivered electrodes with much finer structure exhibiting homogeneous distribution of phases, larger quantity of micro pores and suitable content of Ni and Mo. Tafel slope of electrodes decreased with increasing the in-flight particles velocities from 71 to 33 mV dec^−1^ in 30 wt.% KOH. However, beyond a critical threshold in-flight velocity and temperature of particles, electrodes started to exhibit larger globular pores and consequently reduced catalytic performance and higher Tafel slop of 36 mV dec^−1^ in 30 wt.% KOH. Despite slightly lower electrochemical performance, the electrodes produced with highest plasma gas flow and energy showed most inter-particle bonded structure as well as highest stability with no measurable degradation over 47 days in operation as HER electrode in 30 wt.% KOH. The Raney-type Ni–Mo electrode fabricated at highest plasma gas flow rate and input plasma power has been tested as HER electrode in alkaline water electrolyzer, which delivered high current densities of 0.72 and 2 A cm^−2^ at 1.8 and 2.2 V, respectively, representing a novel prime example of HER electrode, which can synergistically catalyze the HER in alkaline electrolyzer. This study shows that sluggish alkaline HER can be circumvented by rational electrode composition and interface engineering.

## Introduction

Hydrogen has attracted a lot of attention as a clean energy carrier, due to growing pressure on emissions and depleting reserves of fossil fuel. Alkaline water electrolysis (AWE) is one of the most mature and widely used electrolysis technologies for hydrogen production due to the inexpensive non-precious metal electrodes, low cost components and high durability^1–4^. However, AWE operate at significantly lower current densities compared to proton exchange membrane water electrolysis (PEMWE). This can be due to this reason that not only the oxygen evolution reaction (OER) at the anode is a sluggish reaction in AWE, which is true in PEMWE as well, but also hydrogen evolution reaction (HER) at the cathode side is also considered to be sluggish and slow reaction in the alkaline condition^5–8^. Due to this reason, expensive platinum-based catalysts are still the most active catalysts in the alkaline conditions^6,8^. Slow reaction rate of HER in alkaline solution arises from the additional water dissociation step, which releases protons for the subsequent reactions^8^. In particular, in alkaline medium, the HER kinetics involve two steps (Eqs. 1–3): water dissociation into the a hydroxyl ion (OH^−^) and an adsorbed hydrogen atom (H_ads_) called Volmer step, accompanying with the association of adsorbed hydrogen into molecular hydrogen through Heyrovsky or Tafel step^8,9^.

1 $${\text{Volmer:}}\,{\text{ H}}_{{2}} {\text{O }} + {\text{ M }} + {\text{ e}}^{-} \to {\text{ MH}}_{{{\text{ads}}}} + {\text{ OH}}^{-}$$

2 $${\text{Heyrovsky:}}\,{\text{ H}}_{{2}} {\text{O }} + {\text{ MH}}_{{{\text{ads}}}} + {\text{ e}}^{-} \to {\text{ H}}_{{2}} + {\text{ M }} + {\text{ OH}}^{-}$$

3 $${\text{Tafel:}}\,{\text{ MH}}_{{{\text{ads}}}} + {\text{ MH}}_{{{\text{ads}}}} \to {\text{ H}}_{{2}} + {\text{ 2M}}$$

The catalyst role is to greatly increase the rate of all reactions. However, for Pt-based catalysts, which are known to be the most promising catalysts for HER, the catalytic performance and kinetics are hindered by the slow rate of water cleavage in the Volmer step. Due to this reason the catalytic activity of Pt in alkaline media is 2–3 times lower than that in acidic solution^8^. Therefore, the development of highly active electrocatalysts with a significantly hastened Volmer step is highly demanded^9^. The catalyst at the same time should be cost effective. Currently many scientific reports therefore focus on the development of more efficient and inexpensive HER catalysts for the cathode side to improve the performance of AWE electrolyzer^10–13^. Several non-precious metal-based materials, such as transition-metal chalcogenides^14^, carbides^15^ and metal alloys^16^, have been widely studied and characterized as HER electrodes. However, the catalytic activity of these Pt-free based HER catalysts is inferior to the state-of-the-art Pt due to very slow rate reaction of Volmer Step on these catalysts. It has been found that with the introduction of Ni into the Pt lattice, HER performance of Pt-based catalysts drastically increases in alkaline condition, which is due to the capability of Ni to cleave the H–OH bonds facilitating the Volmer step, while Pt facilitates adsorption and combination of the generated hydrogen intermediates to form H_2_ molecules^17^. However, to be cost-effective, it is necessary to completely replace Pt with non-precious metal-based elements, which can easily adsorb and desorb hydrogen to accelerate the Tafel or the Heyrovsky reaction step. It is known that molybdenum (Mo) atoms have outstanding adsorption properties towards hydrogen^9^. Therefore, Raney-type Ni–Mo electrocatalysts can be favorable alternative to efficiently lower the activation energy barrier of the Volmer step and at the same time facilitate the adsorption and desorption of hydrogen, which in turns accelerate the sluggish HER kinetics in alkaline condition^18^. Although various methodologies have been applied for the fabrication of Raney-type Ni–Mo electrocatalysts, the development of free-standing and binder-free electrodes is of great significance for the practical application of the electrocatalytic HER, since those can offer larger specific surface area for reaction and higher conductivity of the electrode^19,20^. Various processes have been applied for free-standing and binder-free Raney-type Ni–Mo based HER electrode fabrication: chemical vapor deposition (CVD)^21^, electrodeposition^22^, plasma spray^7,23–25^ and other thermomechanical method^26^. Among all these processes, plasma spray has been proved to be a promising process for electrode fabrication since it is a rapid prototyping surface modification process, which can endow the surface with desired function and structure in very short time (deposition rates are typically 10 µm cm^−2^ s^−1^)^7,23–25^. In addition, plasma spraying is highly suitable to prepare electrode on large surface areas, e.g. on multi-square meters in a single run, which makes this technology appealing for industrial large-scale production. Plasma spraying can be done under controlled atmosphere e.g. in soft vacuum called vacuum plasma spraying (VPS) or in air referred at atmospheric plasma spraying (APS). The former enables to obtain denser and more uniform coatings with virtually no oxide inclusions^7^, but at higher equipment and running costs compared to APS. As it is essential to avoid using noble metals to keep overall cost of electrolyzer down, it is also imperative to keep electrode production cost low too^7^. However, spraying of metallic particles by plasma in air may lead to their excessive oxidation resulting in passivation and lower electrode performance. The quest is to limit this oxidation by modifying APS operating parameters and attain homogenous coating^27^. Therefore, this work focused on the influence of oxidation and morphology changes of electrodes by applying different energy level and plasma gas flow rate during electrode fabrication using APS on their HER performance.

Five electrodes with different microstructure and elemental distribution were obtained by controlling the input power and the gas flow rates of plasma, which can jointly dominate the temperature and velocity of the powder particles in-flight. NiMoAl was used in the form of powder as a precursor for fabrication of Raney-type Ni–Mo electrodes. Before the electrochemical test, aluminide phases were removed from the electrodes using chemical activation in order to fabricate the Raney-type Ni–Mo and increase the porosity and surface area. The correlation between electrode microstructure and their electrochemical performance was extensively studied using various physical characterization techniques and electrochemical characterization in three-electrode configuration in 30 wt.% KOH solution. To show the relative merits of Raney-type Ni–Mo electrodes as HER electrocatalysts in practical application, the HER electrode with high performance in terms of onset potential and Tafel slope and also highest durability is tested as a cathode electrode along with the APS-based Raney-type Ni as an anode in AWE operated in 30 wt.% KOH.

## Results and discussion

**Figure 1:**
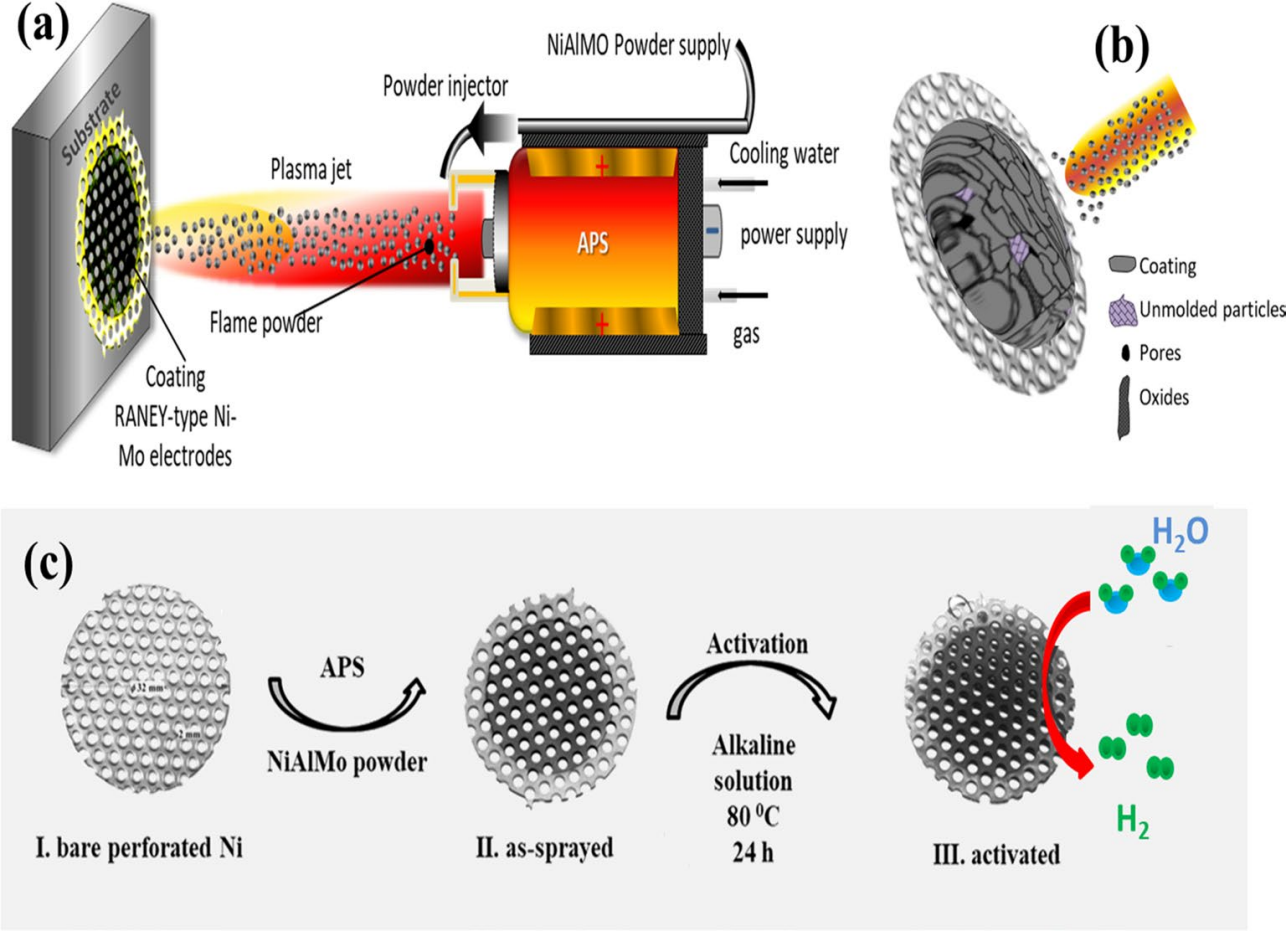
(**a**) Schematic illustration of APS coating of Raney-type Ni–Mo electrode on perforated nickel sheet. (**b**) Schematic structure of a thermal sprayed coating. (**c**) Coating process and chemical activation of Raney-type Ni–Mo electrodes.

Overall APS process for the fabrication of Raney-type Ni–Mo electrodes is shown in Fig. 1. Powder of NiAlMo (44/39/17 in wt.%) with the average particle size of 25 µm is used as a feedstock (see, supplementary information (SI), Figure S1). Briefly, in plasma spraying (Fig. 1a), the powder was injected through injection nozzles into the plasma jet, where particles were accelerated and heated due to momentum and heat transfer between plasma and particles, and the quasi or fully molten (Fig. 1b) particles impacted on the substrate surface followed by flattening, solidification and consolidation to form electrode coating. In this work, five HER electrodes are prepared using NiAlMo powder by APS in a manner that the in-flight velocities and thus inflight dwell times of NiAlMo powder in the plasma were varied by changing plasma conditions, namely plasma gas rate and input plasma power in the range of 42–124 L min^−1^ and 38–59 kW, respectively, Table S1 of SI. This allowed obtaining different oxidation degree and morphology of electrodes. The processing conditions were correlated with electrodes microstructure, their surface morphology and their electrochemical performance. As can be seen in Fig. 1c, after coating, all cathodes were activated in a mixture of 30 wt.% KOH and K-Na-Tartrate-Tetrahydrate solution for 24 h at 80 °C. This activation led to de-alloying and leaching of aluminide phases, forming pores and assisted in achieving high specific surface area. Samples before KOH activation are named as No. 1-BA, No. 2-BA, No. 3-BA, No. 4-BA and No. 5-BA, where BA stands for before activation. Samples after KOH activation are named as No. 1, No. 2, No. 3, No. 4 and No. 5, which correspond to the samples fabricated with the lowest, low, medium, high and the highest gas rate and input plasma power, respectively, (for detailed experimental procedure, please see experimental section).

**Figure 2:**
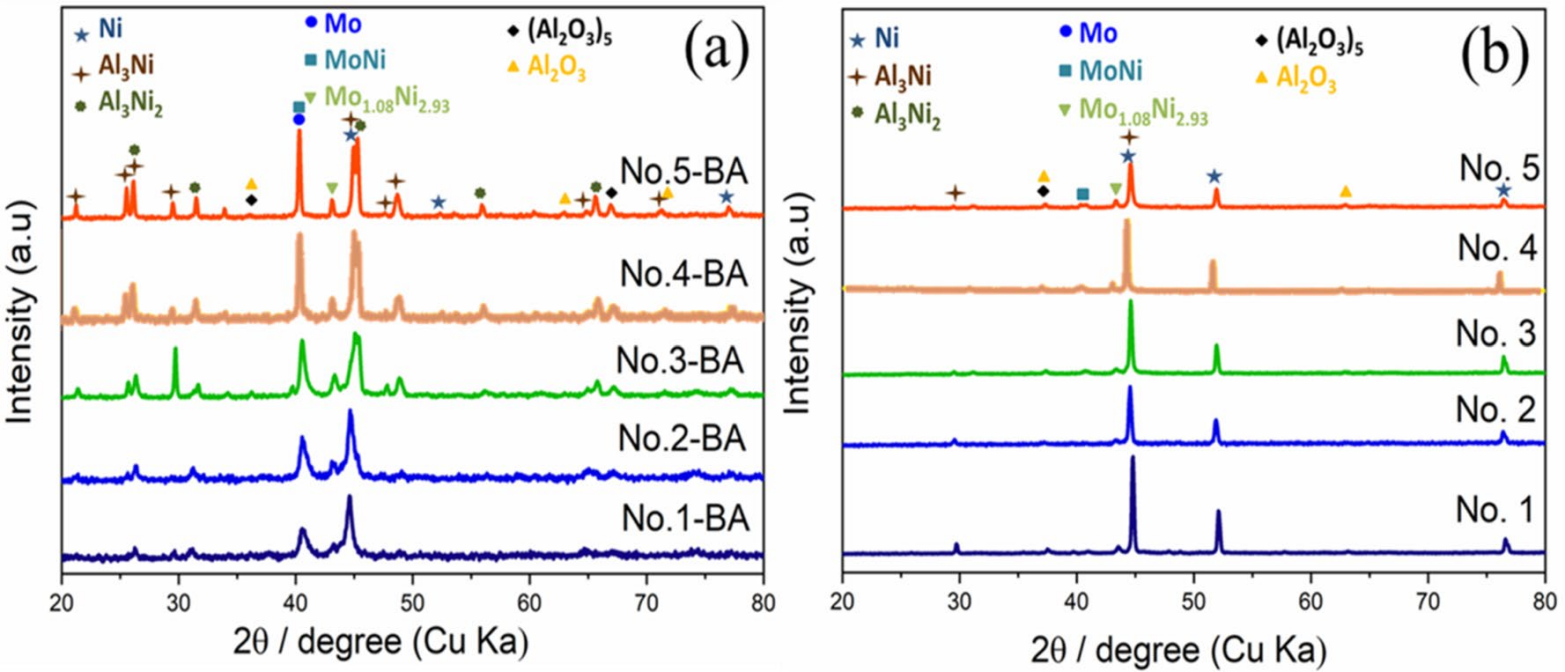
XRD patterns of APS-based Raney-type Ni–Mo electrodes (**a**) before and (**b**) after activation.

Aiming to understand the effect of plasma spray parameters on the phase composition of the produced APS-based electrodes, X-ray powder diffraction (XRD) analysis was carried out. Figure 2 shows the recorded XRD patterns of all APS electrodes before and after KOH activation. As can be seen in Fig. 2a, before activation the XRD results of all the as-sprayed coatings deposited with different plasma power and flow rate exhibit mixed phases with almost similar peaks and different intensities indicating the formation of crystalline composite coatings. In the range of 43°–44° angle, several close lying diffraction peaks of the Al_3_Ni and Al_3_Ni_2_ phases are reported, while the distorted fcc Ni phase is also expected to show a diffraction peak at this angle. Thus, all these phases can be responsible for the observed diffraction peak at this angle. Therefore, the crystalline peaks appeared in the XRD patterns of the as-prepared coatings are associated to Ni (PDF No. 65-2865), Al_3_Ni (PDF No. 00-002-0416), Al_3_Ni_2_ (PDF No. 01-083-3987), Mo (PDF No. 00-042-1120), MoNi (PDF No. 00-048-1745) and Mo_1.08_ Ni_2.93_ (PDF No. 01-071-9764) phases. Some other peaks attributed to the (Al_2_O_3_)_5_ and Al_2_O_3_ are also observed. As can be seen in Fig. 2a, the intensity of peaks increases suggesting the increase in the grain size by increasing the plasma power^28,29^. The major XRD peaks of electrode No. 1-BA fabricated at lowest flow rate and the lowest plasma power is attributed to the Ni and Al_3_Ni phases. However, increase in the extent of crystalline phases was observed by increasing the flow rate and the plasma power. This can be attributed to the different factors such as, high heat input, the degree of powder melting, the extent of oxidation and heat accumulation in the coatings during the plasma spraying process^30^.

Figure 2b, shows the XRD profiles of KOH activated electrodes. All electrodes show similar prominent peaks corresponding to the distorted Ni suggesting the removal of some compound phases including Al_3_Ni_2_ and partially Al_3_Ni during KOH activation. Apart from distorted Ni phase and Al_3_Ni, peaks for the MoNi, Mo_1.08_Ni_2.93_, Al_2_O_3_ and (Al_2_O_3_)_5_ are also observed^7^. It is worth mentioning that, after KOH activation the main peak intensities centered at 44° start decreasing from electrode No. 1 to electrode No. 5. The higher peak intensity of No. 1 can be due to the overlapping of Al_3_Ni and distorted Ni peak suggesting that some aluminide phases are not accessible to the KOH solution during activation for the electrode fabricated at lower flow rate and lower plasma power. In addition the peak for Mo is found to be drastically decreased after activation suggesting removal of unreacted Mo during activation.

**Figure 3:**
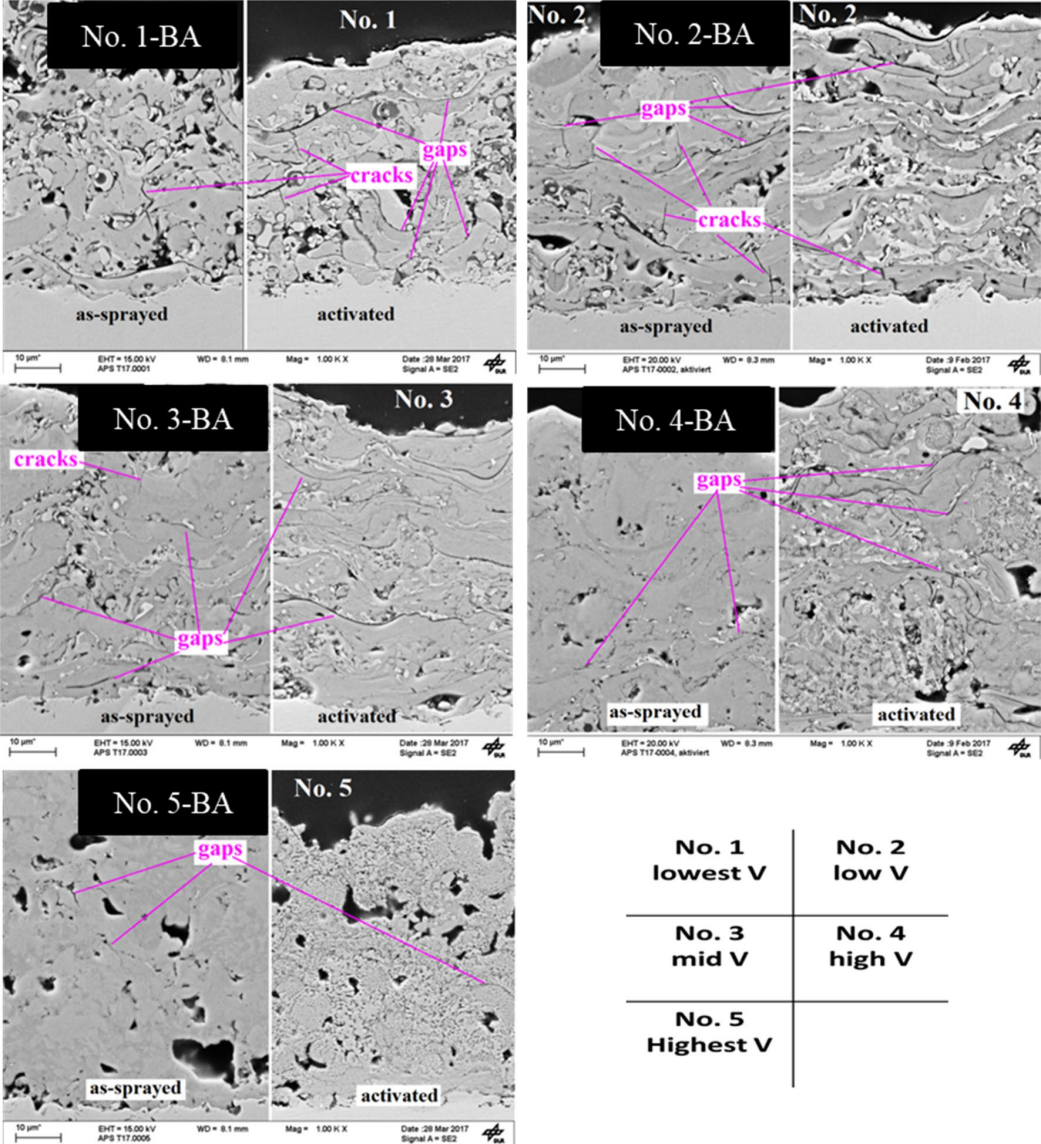
SEM images of all APS-based Raney-type Ni–Mo electrodes before and after activation.

The microstructure of the electrode before and after KOH activation was analyzed by scanning electron microscope (SEM). Figure 3 presents the cross-sectional SEM images of all the five as-prepared and activated samples fabricated at different plasma power and flow rate. Figure 3 shows that electrode No. 1 structure fabricated at lower plasma power and lower flow rates with the lowest in-flight particle velocity exhibited a large number of entrapped quasi-spheres, which could be due to re-solidification of particles in-flight after their melting, evaporation and entrainment in the plume of the plasma. With the increase in the plasma power and flow rate, the in-flight velocity of particles were gradually increased (electrode No. 1 to No. 5) and coating composed of better melted and adhered lamella leading to denser and more homogeneous structure with fewer inter-lamellar gaps and cracks. By increasing the applied higher energy the in-flight velocity of NiAlMo powder increases leading to shorter flight time of particles in the hot plasma. On one hand this leads to higher momentum and impact energy of particles and on the other hand reduced the segregation of alloying elements (NiAlMo) that can occur in the molten particles during their flight due to the difference of density and surface tension of the alloying elements. This indicates that higher velocity led to improved consolidation and mechanical integrity of electrodes and segregation of phases, as appears as different shades, can be also avoided. Compared to electrode No. 4, electrode No. 5 prepared with the highest plasma power and highest flow rate, similar trend of improved inter-lamellar cohesion, interlayer bonding and reduced segregation was observed. However some larger globular pores in the range of 1–10 µm could be seen in electrode No. 5. As it is reported, beyond a critical velocity and temperature, the molten metallic particles with low viscosity tend to form splashed and fragmented splats, which can lead to formation of globular pores^30^, which is expected to be the case for sample No. 5. As can be seen in Fig. 3, compared to the as-prepared catalyst layer, activated samples showed porous sponge-like structure with several gaps between layers. The sponge structure tends to increase for coatings produced with higher velocity whereas the inter-splat gaps tend to decrease. This can be attributed to the removal of aluminide species such as Al_3_Ni_2_ and partially Al_3_Ni and unreacted Mo during the KOH activation. Electrodes produced with higher velocity appear to exhibit higher intra-splat leaching instead of inter-splat removal of phases and vice versa. For example, electrode No. 5 shows most spongy porous structure with highly fine and homogenous microporosity and strongly reduced cracks and gaps between the layers. Comparatively, electrodes fabricated at lower plasma power and lower flow rate were seriously deteriorated during activation and contained visible gaps and cracks in their structure. From SEM and XRD results, it can be suggested that with spray conditions leading to lower velocity of powder, the segregation of phases occurs during in-flight, which appear in inter-splat region in electrodes. Consequently it can be expected that there is lower content of these aluminide phases intra-splat. Therefore, leaching for electrodes produced with high velocity predominantly occurs intra-splat leading to higher degree of spongy structure whereas for electrodes fabricated with low velocity inter-splat leaching was pronounced causing deterioration in cohesion.

Elemental composition of APS-based Raney-type Ni–Mo electrodes before and after activation was investigated by SEM/EDX and EPMA. As can be seen in Table S2 of SI, all as-prepared electrodes contain oxygen. Presence of oxygen in all as-prepared electrodes can be due to the partial oxidation of the NiMoAl powder during the fabrication. The O fraction of the as-prepared electrodes before activation decreased from electrode No. 1 to No. 5 as their in-flight velocity was increased during fabrication. The Al fraction was increased from 28.47 wt.% for sample No. 1-BA to 37.41 wt.% for sample No. 5-BA by limiting in-flight loss of Al. Although the raw material of the feedstock consists of 39 wt.% Al, evaporation, oxidation and spray loss jointly cause the lower Al fraction of the as-sprayed electrodes. The variation of Ni and Mo fractions between the as-sprayed samples was much smaller with magnitude of 2%, which is mainly due to the relatively higher melting point of Ni and Mo compared to Al. During the activation, Al components and unreacted Mo are partially etched off forming cavities and pores. As shown in EDX in Figure S2 of SI, elemental mapping images for APS-based Raney-type Ni–Mo electrodes after activation indicate the existence of Ni, Mo, Al and O in all the electrodes. As can be seen in Table S2 of SI, Al is partially leached out after KOH activation; least in electrode No. 1 (Al content 28.47 wt.% before and 25.80 wt.% after leaching) and most from electrode No. 5 (37.41 wt.% before leaching and 11.40 wt.% after leaching). This shows that more Al-based species has been removed after KOH activation for samples fabricated at higher velocity suggesting the presence of more accessible and also more available Al rich phases such as Al_3_Ni_2_ and Al_3_Ni, which are less resistant to the KOH. This is in agreement with the XRD results where the higher intensity of aluminide phases was observed for the electrode No. 1 fabricated at lower plasma parameters and lower intensity of aluminide phases was observed for the electrode No. 5 fabricated at higher plasma parameters. Mo content also decreased after KOH activation compared to as prepared sample. This is in agreement with XRD, which shows decrement in Mo peak intensity after activation, suggesting removal of unreacted Mo after KOH activation. Moreover, after KOH activation O content of all samples increased; least increase was for sample No. 1 (5.94 wt.% before and 8.5 wt.% after leaching) and most for No. 5 (2.01 wt.% before and 11.1 wt.% after leaching). This increase of O content after activation can be due to the OH^−^ adsorption during the KOH activation process and eventually formation of Ni(OH)_2_ and also catalyst surface passivation^7,23^. Therefore, after activation the electrodes that exhibited higher loss of Al with higher Ni content also exhibited higher increase in the porosity, surface area and also higher O fraction gain.

**Figure 4:**
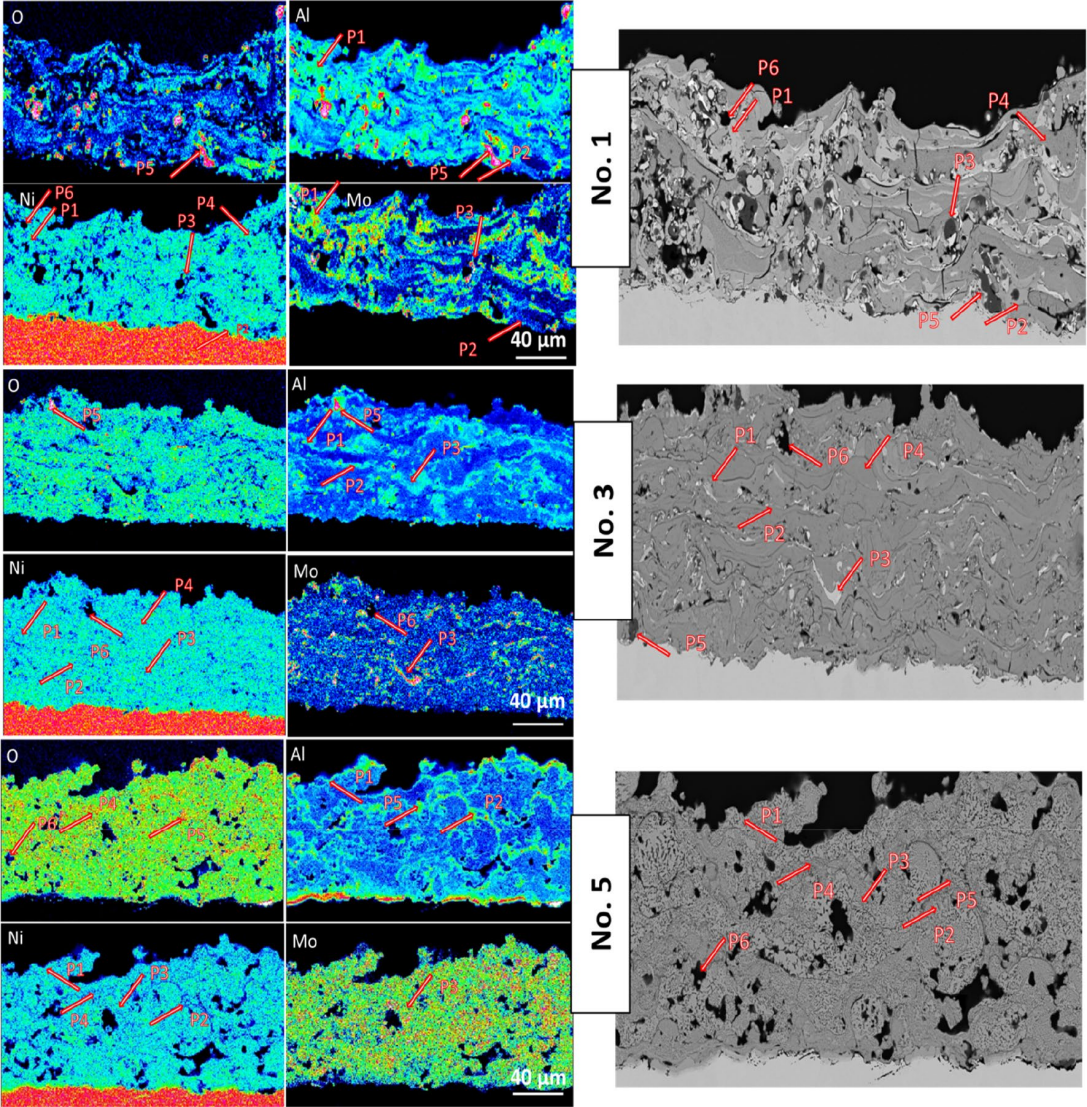
EPMA mappings of electrode No. 1, No. 2 and No. 3, showing the elemental distribution of Ni, Al, Mo and O.

As shown in Table S3 of SI and Fig. 4, six spots of electrodes No. 1, No. 3 and No. 5 were checked by EMPA. As shown in the SEM and its EPMA mapping in the Fig. 4, for all samples P1-P4 present Ni phase, mixture of alloy phases composed of Ni, Al, Mo and the oxide phases. P5 is mostly the Al_2_O_3_ and (Al_2_O_3_)_5_ phase and P6 indicates the porous phase. It can be seen at spot P1 and P2 where the green colour for Al is overlapping with the blue colour for Ni indicating the presence of aluminide phase. However, some O and Mo also can be detected in these spots. However, spot P3, where the blue colour for Ni is overlapping with the red colour for Mo suggests the presence of Ni and Mo alloys. However, the trace of some Al and O also can be detected on this spot. P4 mostly presented the distorted Ni. It can be seen that the oxygen spots (dark pink) is higher in the electrode No. 5 compared with two other electrodes. This can be seen from the EPMA of electrode No. 5, where more O with dark pink color is distributed all over the Ni-based sample. This can be due to the formation of Ni(OH)_2_ during activation and also catalyst surface passivation. Therefore, after activation No. 5 with higher loss of Al but having higher Ni content exhibited higher O fraction gain due to formation of Ni(OH)_2_ and surface passivation. According to the EPMA images, P1-P2 spots mostly found to be trapped between layers and gaps in electrode No. 1 and No. 3 prepared at low and medium plasma parameters. This made removal of trapped aluminide phases much more difficult during KOH activation, which leads to their low porosity. However, it can be seen from EPMA images that higher porosity is observed for electrode No. 5, where more aluminide rich phases are removed and Mo in the form of Ni and Mo alloys are homogeneously dispersed all over the electrode backbone^7^. The presence of Al_2_O_3_ and (Al_2_O_3_)_5_ in all electrodes indicated by P5 can be observed by the green color in the EPMA images in the Al mapping, which overlaps with the space covered with O (dark pink) in Fig. 4. It can be seen in Fig. 4, that more agglomerated Al_2_O_3_ and (Al_2_O_3_)_5_ can be found in the electrode No. 1 and No. 3 than electrode No. 5 indicating the more homogeneous elemental dispersion in the electrode No. 5. The variation between different spots by varying the plasma parameters from electrode No. 1 to electrode No. 5 can be also seen from the related SEM images. It can be seen that the variation of grey value between these spots was found to be affected by the amount of Ni that with high fraction of Ni the phase turned to be bright grey. This result is in agreement with the XRD, SEM and EDX analysis.

**Figure 5:**
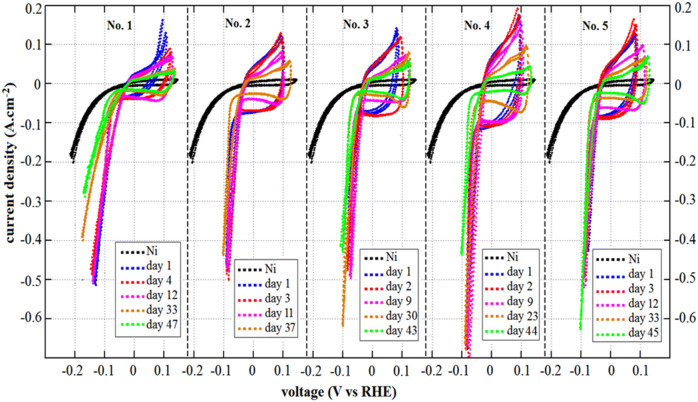
CV curves of all APS-based Raney-type Ni–Mo electrodes within a potential window between − 0.21 V and 0.15 V (vs. RHE) obtained during durability test conducted at a constant current of 2 A for 5 weeks.

The electrochemical activity of all activated samples is scrutinized for HER. CV curves at the scan rate of 5 mV s^−1^ within a potential window between − 0.21 V and 0.15 V (vs. RHE) are obtained in 30 wt.% KOH solution for all electrodes during the stability test, which is performed at a constant current of 2 A for 47 days. As shown in Fig. 5, all APS-based Raney-type Ni–Mo electrodes show better HER activity than the pure perforated Ni film as a reference. As the flow rate and the plasma power increase, the HER performance improved from electrode No. 1 to No. 4. The overpotential of 100, 90, 85 and 80 mV were obtained at the current density of − 200 mA cm^−2^ for the electrode No. 1, No. 2, No. 3, and No. 4, respectively. However, a slight decrease in performance for electrode No. 5 (overpotential of 82 mV at the current density of − 200 mA cm^2^) was observed compared with that of No. 4.

As can be seen from Fig. 5, the area under the CV curves starts to increase from electrode No. 1 to electrode No. 4, which can be related to higher electrochemical active surface areas (ECSA) of electrodes fabricated at higher flow rate and the higher plasma power. However, despite the presence of much finer micropores in electrode No. 5, the area under the CV curve slightly decreased compared to that of electrode No. 4. This decrement can be due to presence of more macropores in its backbone compared to that of electrode No. 4, shown in SEM images. It is reported that the pore structure of electrode is a significant element in determining electrochemical performance^31^. Pores of different sizes (micro-, meso- and macropores) play different roles in contributing to double-layer (DL) capacitance, which directly affect the ECSA and electrochemical performance^31^. In spite of micropores, which are responsible for most of the specific surface area, macropores can cause the effective loss of active surface area and my thus not contribute much to the DL capacitance^31^. Therefore, the electrochemical double-layer capacitance (C_dl_), which is proportional with the ECSA was further used to estimate the effective surface areas by CV test in the non-faradaic region, 0.3–0.5 V, as shown in Figure S3 of SI.

As shown in Figure S3 of SI, the values of C_dl_ increased from No. 1 to No. 4 with the value of 31, 46, 63, and 167 mF cm^−2^ for No. 1, No. 2, No. 3 and No. 4, respectively, and then slightly decreased to 120 mF cm^−2^ for No. 5 indicating that the electrode No. 4 possesses the highest effective surface area for HER. This also explains the slight activity loss associated with the formation of macropores and loss of some active area in the electrode No. 5. For all APS-based Raney-type Ni–Mo electrodes, it can be observed that obvious changes of the CV shape occur during the 47 days. This may be due to the losses of catalyst materials and/or the irreversible chemical and structural restructuring of the reaction interface^32^. During the cycling the active sites can be leached out with the aluminide phases, which in turn can decrease the electrochemical active surface area resulting in decrease of overall HER performance. However, all five samples exhibited different degradation behaviour under the durability test over the 47 days (Fig. 5). Among all the samples, sample No. 1, prepared at the lowest flow rate and plasma power showed the highest degradation with increasing overpotential by 30 mV at current density of − 200 mA cm^−2^ during 47 days, while others show much lower potential requirement for achieving the same current density after the durability test. The most likely degradation mechanism is the detachment of delimitated layers. This result is in agreement with the SEM images in Fig. 3, which show that electrode No. 1 was the most fragile sample with a lot of gaps, cracks and un-molten particles between layers, which can cause poor connection and low robustness. This fragile structure of electrode No. 1 leads to delamination of catalyst layer from the substrate during the harsh condition of electrochemical testing in 30 wt.% KOH at high constant current density of 2A. Due to this reason, a lot of dust-like black sediments were observed in the electrolyte solution after several days of test for electrode No. 1. However, other samples presented moderate increase in overpotential after a slight decrease during the first several days, which is most likely due to the initial activation of electrodes in KOH solution under the constant current. It is reported that the aluminide components cannot be completely removed during the first chemical activation in KOH^7,23^. However, during the electrochemical operation under the constant current, presence of the alkaline electrolyte possibly drives reactivation of the residual Al components from the electrode backbone to induce a temporary improvement of the active area. However, after a few days consequent deterioration was observed for all samples over the entire test (increasing the overpotential), which can be due to delamination of Ni as an active site along with the leaching of aluminide phases. But the deteriorating rate was different for various electrodes. Among all the APS-based catalysts, electrode No. 5 is found to be the most stable electrode, which showed only 2 mV increment in overpotential at current density of − 200 mA cm^−2^ after 45 days. These can be due to the presence of less aluminide phases in its backbone and lower degree of loss of active sites during cycling. According to the results of EPMA, the interlayer of electrodes mainly consisted of aluminide phases. As it has been discussed earlier, due to the more homogeneous structure of electrode No. 5 with fewer inter-lamellar gaps and cracks, more aluminide phases were leached out by KOH activation and Mo is homogenously dispersed all over the electrode structure likely in the form of Ni and Mo alloys. As a pore former, Al brings no contribution to the anti-corrosion ability, but to enlarge the active site after being leached out. Moreover, the enrichment of Al on the interface will cause peeling-off the coating, leading dust-like sediments and degradation of electrode performance. Conversely, the existence of Mo gives the electrode higher resistance to electrochemical corrosion^33^. Therefore, with homogenous distribution of Mo and presence of lower amount of aluminide phases, sample No. 5 endowed the lowest deterioration of overpotential among all other APS-based electrodes with more gaps and cracks enriched by aluminide phases.

**Figure 6:**
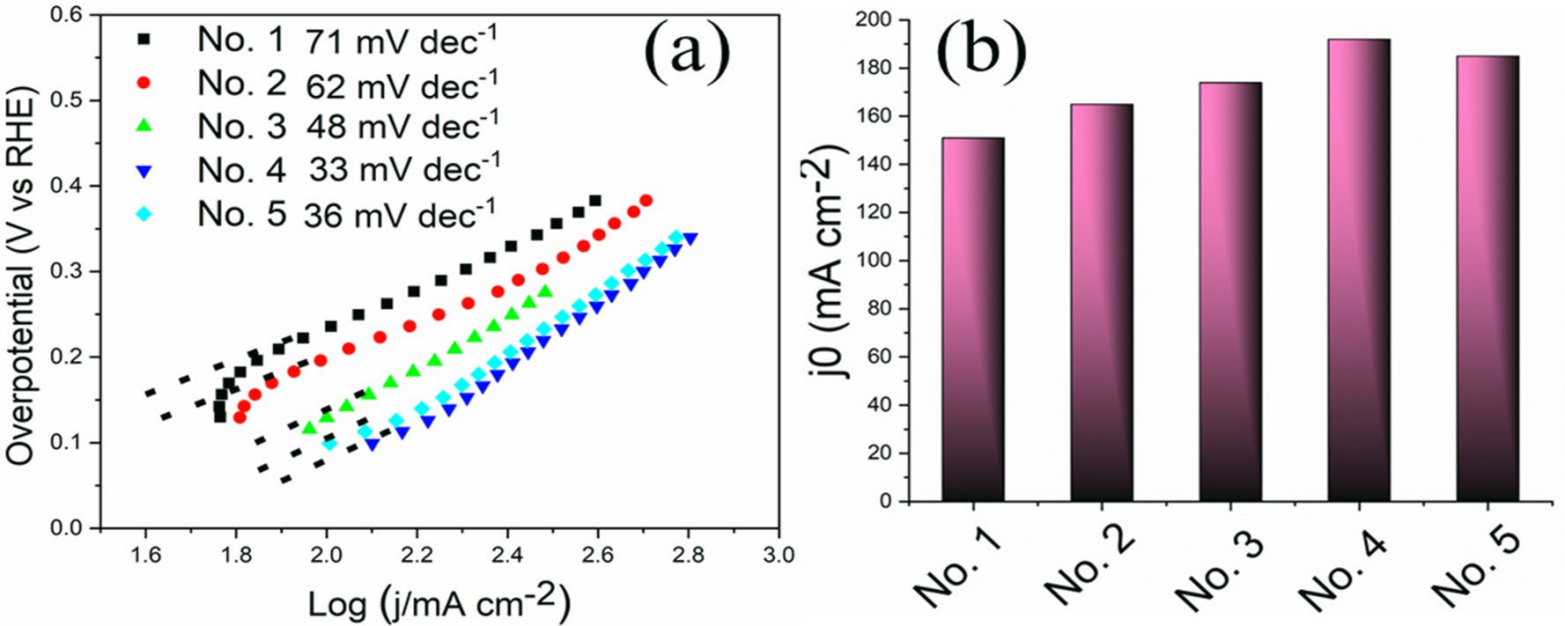
(**a**) Tafel plots of APS-based Raney-type Ni–Mo electrodes and (**b**) Exchange current densities of APS-based Raney-type Ni–Mo electrodes determined by extrapolating the Tafel plots to the x axis.

**Figure 7:**
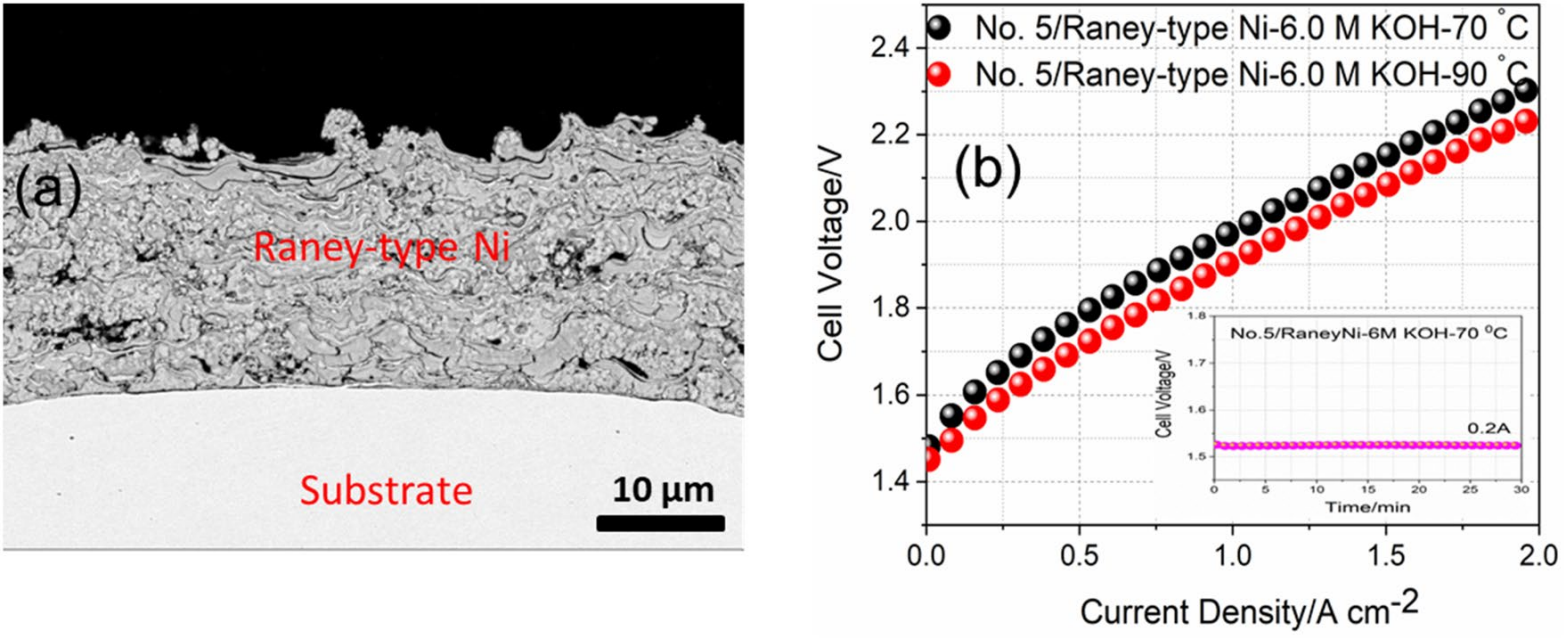
(**a**) SEM images of activated Raney-type Ni fabricated by APS, which is used as an anode side alkaline membrane based water electrolyzer. (**b**) Current density versus applied voltage plot of the alkaline water electrolyzer using electrode No. 5 and APS-based Raney-type Ni as anode and cathode, respectively, in 30 wt.% KOH at 70 and 90 °C (inset shows the pre-activation of cells using chronoamperomerty (CA) at constant current of 0.2A for 30 min at 70 °C).

In order to further assess the intrinsic HER activities of the catalysts, the Tafel plots of all different samples are analyzed. Tafel slope can be used to highlight the advantages of the modified APS process and probe the effects of different flow rate and plasma power, which leads to formation of different micro and macro pores and different elemental composition during electrode fabrication on the HER rate-determining steps. Note that for hydrogen evolution in alkaline solutions on a metallic (M) electrode, the mechanism typically involves three major reactions of Volmer reaction with the Tafel slope of 118 mV dec^−1^, Heyrovsky reaction with the Tafel slope of 40 mV dec^−1^, and Tafel reaction with the Tafel slope of 30 mV dec^−1^. From the potential-current data the cathodic Tafel slopes are calculated and this kinetic data is presented for each electrode in Fig. 6a. The Tafel slope was found to decrease from electrode No. 1 to No. 4 with the value of 71, 62, 48 and 33 mV dec^−1^ for No. 1, No. 2, No. 3 and No. 4, respectively, and then slightly increase to 36 mV dec^−1^ for electrode No. 5. As can be seen in Fig. 6a, in comparison with all other electrodes, sample No. 1 shows the highest Tafel slope of 71 mV dec^−1^, indicating that the mostly Volmer reaction and water dissociation is the rate-determining step for the electrode No. 1. This result is in agreement with higher overpotential of electrode No. 1 compared with other electrodes. Since Ni is reported to be responsible for water cleavage and the Mo is responsible for the hydrogen adsorption and recombination^6^, due to lower amount of Ni (42 wt.%) in electrode No. 1 than other electrodes and also comparatively lower microporosity in its backbone, which limits electrolyte accessibility to the active sites, Ni is most probably not fully available to dissociate the water molecule. However, other electrodes initially presented Tafel slope in the range of ~ 30–60 mV dec^−1^, which can be assumed that the desorption reaction (the Heyrovsky or the Tafel step) is the rate determining step. As can be seen in Table S2 of SI, with increasing the flow rate and plasma power, the Ni and Mo contents start increasing and decreasing, respectively, from electrode No. 1 to No. 5. It has been already observed that the electrode microstructure of all catalysts has been optimized compared to sample No. 1. Therefore, due to higher and optimum amount of Ni in these electrodes and increasing the porosity, which facilitate the accessibility of electrolytes to the active sites, the water molecule dissociation is facilitated compared with that of electrode No. 1. However, as can be seen in Fig. 6a, the lowest Tafel slope has been achieved for the Electrode No. 4, which can be due to its high electrochemical surface and appropriate amount of Ni and Mo in this electrode. Electrode No. 5 shows slightly higher Tafel slope than that of No. 4. Thus the electrocatalytic activity is improved to some extent by increasing the flow rate and plasma power. Exchange current density (j°) indicates the intrinsic catalytic efficiency of electrocatalysts, which can be determined by extrapolating the Tafel plots to the x axis. The j_0_ can be assessed by assuming overpotential (η) was zero with the Tafel equation. As shown in Fig. 7b, the j° value of No. 4 electrode shows the highest value of 192 mA cm^−2^, compared with that of 151, 165, 174 and 185 for No. 1, No. 2, No. 3 and No. 5, respectively, which is in agreement with its lower overpotential compared with all other electrodes. These results reveal the origin of the excellent HER activity of the electrode No. 4.

As it has been discussed in alkaline media, the HER pathway could be through the Volmer–Heyrovsky process or Volmer–Tafel pathways. Both pathways involve two steps. In the first step, the adsorption of H_2_O molecule and electrochemical reduction of adsorbed H_2_O into adsorbed OH^−^ and H atom can take place. However, in the second step, desorption of OH^−^ to refresh the surface and formation of H adsorbed intermediate for H_2_ generation take place. It is reported that in the NiMo based catalyst, Ni is responsible for the first pathway and Mo is responsible for the second pathway^17,9^. However, the OH^−^, which comes from KOH solution and also generated by H_2_O splitting can attach to the Ni sites during passivation and also during electrochemical reaction. This can in turn occupies the sites for H atom adsorption, causing inefficient release of OH^−^ and blocking the active catalytic sites. Regarding the oxides, most of the transition metal oxides and hydroxides including oxides and hydroxides of Ni exhibit intrinsically low electrical conductivity and unfavorable hydrogen adsorption desorption capability^34^, which hinder their use as electrocatalysts for the HER in alkaline media. Their low conductivity enables effective charge transfer in the electrocatalytic process to occur on the surface of electrocatalysts. Therefore, Ni oxide and hydroxides formed during the passivation and electrochemical reactions^35,36^, are not specifically active for HER due to low conductivity and the lack of H adsorption sites. However, we have to consider that HER activity is not only influenced by conductivity. The HER performance of electrodes can be enormously influenced by several parameters, porosity, conductivity and active sites, which require an optimization. Since the surface area, number of active sites and conductivity varies significantly as a function of plasma parameters during coating process, the trade-off among them should be discussed fully with the plasma parameters for better understanding of electrochemical properties. In this present study, we observed an increase in ECSA and the HER activity of electrode No. 4 compared with that of electrode No. 5, which has a higher Ni content and lower Al species than those of electrode No. 4. These improved properties can be attributed to a more homogeneous structure and lower oxidation degree, which can result in higher conductivity of electrode No. 4 compared with that of electrode No. 5. Since it is reported that in Mo–Ni-based alloy electrocatalysts Ni is the appropriate element to dissociate water molecule and Mo has outstanding adsorption properties towards hydrogen, thus, the appropriate Ni and Mo content in electrode No. 4 compared with that of electrode No. 5 can be another reason for its HER activity. Regarding the oxygen content and its influence on conductivity it is noted that electrodes No. 1 to No. 3 have lower oxygen content than electrode No. 4, however they have much lower HER activity which can be due to the lower porosity, higher amount of Al and inappropriate Ni and Mo content, which are obviously more important parameters for HER performance. Therefore, Electrode No. 4 due to most favorable trade-off between key factors such as its porous structure with appropriate micro-/macropore distribution, amount of active Ni and Mo species and its optimized level of oxidation degree shows the highest HER activity among all APS-based Raney-type Ni–Mo electrodes. As shown in Table S4 of SI, the best HER catalyst in terms of activity obtained in this work shows higher performance compared to other NiMo catalysts reported in other literatures. This indicates that in this work optimizing APS parameters successfully resulted in fabrication of highly active HER catalyst.

Since beside HER activity, durability and the constant performance of HER catalysts over the time is the most important factor for the practical use of catalysts, the very stable electrode No. 5 with slightly lower HER activity than the electrode No. 4 can be considered as the ideal practical HER electrode candidate in the real alkaline based electrolyser. The performance of the single cell based on the cell configuration with sample No. 5 as the HER electrode for the cathode and the Raney-type Ni as the OER electrode for the anode and membrane (Dapozol) in 30 wt.% KOH at 70 and 90 °C in terms of polarization curves has been assessed and is shown in Fig. 7. Figure 7a shows the SEM image of the Raney-type Ni with highly porous sponge-like structure fabricated by APS process. As shown in Fig. 7b, the membrane electrode assembly (MEA) constructed with electrode No. 5 as a cathode and Raney-type Ni as an anode has been tested in AWE with 30 wt.% KOH at two different temperatures of 70 and 90 °C. As can be seen in inset of Fig. 7b, cell is pre-activated using chronoamperomerty (CA) at constant current of 0.2A for 30 min before recording the polarization curves. The AWE cell exhibited high current densities of 0.56 and 2.3 A cm^−2^ at 1.8 and 2.2 V, respectively, in 30 wt.% KOH at 70 °C. This performance is very close to that of the recently reported performance with the same electrode package in 30 wt.% KOH using the Zirfon separator^37^. However, the cell showed even higher performance with current densities of 0.72 and 2 A cm^−2^ at 1.8 and 2.2 V, respectively at slightly elevated temperature 90 °C, representing a novel prime example of HER catalysts, which can effectively catalyze the HER in alkaline electrolyzer.

## Conclusions

This work develops a rational methodology to attain promising performance from non-noble metal based NiMo alloys as catalysts for the sluggish HER in alkaline electrolytes. This was achieved by fabricating the catalyst layer as binder-free electrodes by atmospheric plasma spraying and with precise control of processing conditions to correlate fabrication parameters with the structure properties and with the performance of the electrodes. It was shown that with increasing the input plasma power and flow rate, powders attained higher velocity and momentum in-flight plasma and the resulting coatings of electrodes exhibited finer and larger quantity of pores and suitable Ni and Mo content after activation resulting in improved HER activity. When APS-based Raney-type Ni–Mo materials tested as HER electrodes in alkaline condition, electrode No. 4, obtained particularly at high flow rate and input plasma powers due to the proper pore distribution and efficient Ni and Mo content along with the lower Al content illustrates lower overpotential of 80 mV for three-electrode system in alkaline electrolyte among all other APS-based Raney-type Ni–Mo materials fabricated at lower flow rate and lower plasma power. Although, electrode No. 5 fabricated at highest flow rate and plasma power slightly shows lower HER performance than electrode No. 4 due to its higher macro porous structure. However, electrode No. 5 exhibits the highest stability with no measurable degradation over 47 days. The high durability of electrode No. 5 can be due to the lower aluminide phases in its backbone and lower degree of loss of active sites during cycling. The AWE with the electrode No. 5 as the HER electrode, Raney-type-Ni as the OER electrode and commercial membrane in 30 wt.% KOH delivers high current density of 2 A cm^−2^ at 2.2 V, delivering a novel prime example of such hybrid electrodes which can synergistically catalyze the HER in basic media.

## Experimental

### Material fabrication

#### APS-based electrode fabrication

The Raney-type Ni–Mo based electrodes are produced by spraying spherical gas-atomized powders of NiAlMo (44/37/19 in wt.%) with the average particle size of 25 µm (Figure S1 of SI) supplied by HC Stack on perforated Nickel sheet with dimension of 32 mm × 0.5 mm and 69% effective area, by APS technique. A Triplex-Pro210 plasma gun from Oerlikon-Metco (CH) is used for APS for which Ar is the primary plasma forming gas and H_2_ and/or He are as secondary gases. NiAlMo powder are fed into the plasma stream, in which the powders melt and are sprayed over the substrate to be coated. Multiple layers are coated to form electrodes with suitable thickness. The thickness of Raney-type Ni–Mo layer was designed to be close to 100 µm, which is empirical value to exhibit high performance. Five HER electrodes are prepared using NiAlMo powder by APS with controlled input power and the flowrate of plasma gas, Table S1 of SI. An exposing time of NiAlMo powder in the hot plasma plume could be varied from case to case to achieve different oxidation state and morphology. In order to understand the correlation between electrode microstructure and surface morphology of the coatings with their electrochemical performance, gas rate and input plasma power were varied in the range of 42–124 L min^−1^ and 38–59 kW, respectively. By applying the 5 sets of operating parameters, distinct velocities and temperatures of the in-flight particles are obtained by AccuraSpray G3 (Tecnar, Canada). Due to the extremely high luminosity of the plasma, the selected measuring point is 170 mm away from the exit of the torch. The optic head of AccuraSpray is horizontally installed to perpendicularly watch the particle at 120 mm. The measured value of velocity and temperature is not the same as the where coatings are sprayed; it nevertheless reveals the difference of particle velocity between the 5 sets of parameters. As displayed in Figure S4 of SI, the five sets of parameters are successfully designed to achieve particle temperature higher than the melting point of NiAlMo alloy which is about  1800 °C; and the achieved particle velocity is varied from 240 to 580 ms^−1^. Due to the difference between the measuring distance (170 mm) and the spraying distance (60 mm) it can be inferred that in all cases the spraying temperature and velocity are higher than the measured value in Figure S4 of SI. Moreover, owing to the increase of plasma gas, the plasma enthalpy is accordingly increased to try to maintain comparable particle temperature between these cases, Table S1 of SI. For example, from electrode No. 1 to electrode No. 2, the input power is increased from 38.1 to 42.1 kW, Table S1 of SI, as a provision to the increased plasma gas. Five coating samples were prepared under different flow rate and input plasma powers, denoted as No. 1, No. 2, No. 3, No. 4 and No. 5, respectively, corresponding to the samples fabricated with the lowest gas rate and the lowest input plasma power to the highest ones (see Table S1 of SI). The electrodes were prepared at different plasma power and flow rate, which the lowest flow rate and lowest input plasma power is applied for fabrication of electrode No. 1 and the highest flow rate and highest input plasma power is applied for electrode No. 5. Before, the electrochemical test, for achieving high specific surface area, all cathodes were firstly activated in a mixture of 30 wt.% KOH and K-Na-Tartrate-Tetrahydrate (≥ 99%, Carl Roth GmbH) solution for 24 h at 80 °C. Less resistant aluminide phases to KOH solution and some unreacted metals was partially removed after chemical activation. The samples before KOH activation are named as No. 1-BA, No. 2-BA, No. 3-BA, No. 4-BA, and No. 5-BA, where BA stands for before activation and samples after KOH activation are named as No. 1, No. 2, No. 3, No. 4 and No. 5. Furthermore, the anode electrode Raney-type Ni based electrode is also produced by spraying powders of NiAl (44–56 wt.%) with the particle size of 45 µm supplied by HC Stack on Punched Nickel sheet by atmospheric plasma spraying (APS) with 30 kW plasma energy.

### Material characterization

X-ray diffraction (XRD) patterns of the coating samples were acquired using an X-ray diffraction (XRD) patterns of the coating samples were acquired using an X-ray diffractometer Siemens D5000 (IMW, Germany) in the Bragg–Brentano geometry with Cu-Kα source operated at 30 kV and 25 mA. In order to fix samples for the further analysis such as Scanning Electron Microscopy (SEM) and Energy Dispersive X-ray Detector (EDX) the samples are made into the resin. The applied resin is the mixture of resin and hardener with the mass ratio of 25:3. After mixing the resin components for several minutes, the bubbles produced during mixing will be forced out with the help of a vacuum pump. The electrode films are placed in the mould and the resin mixture is poured into the mould to cover all the samples followed by drying in ambient temperature for 8 h. Before the physical characterization the resin samples are polished using different grinding size 46 µm, 22 µm, 15 µm, 9 µm and 3 µm to make the surface smoother and clearer. The morphology of the polished resin electrodes was observed with SEM using a ThermoFisher SCIOS microscope operated at an acceleration voltage of 20 kV. The chemical compositions of the samples were determined by the EDX spectrometer/detector from Bruker Company. The obtained electrodes are also investigated with a scanning electron microscopy (SEM: S-570 by Hitachi) equipped with an electron probe micro analysis (EPMA: EMAX-5770 by Horiba). As for an EPMA measurement, the cross sections of the Raney-type Ni–Mo alloy samples were studied for a depth profile.

### Electrochemical characterization

All of the electrochemical measurements were carried out at room temperature using a Zahner Elektrik IM-6ex potentiostat with a developed in-house three-electrode test bench including a reference electrode (reversible hydrogen evolution, RHE, Gaskatel GmbH), a Ni as a counter electrode and the working electrode. The working electrode was fixed in a Plexiglas frame with an effective opening of 4 cm^2^ as a compromise between saving material and easy handling on one side and properties close to those of technical size electrodes on the other side. The electrochemical performance of Raney-type Ni–Mo based electrodes was carried out in in 30 wt.% KOH at 70 °C sealed from air. The produced anode gas and cathode gas is carefully arranged to be safely exhausted to the air. Firstly, cyclic voltammograms (CV) of the working electrodes were measured at scanning rate of 5 mV s^−1^. The electrode was cyclically operated in the potential window in the range of − 0.21 V to 0.15 V vs RHE. In order to get comparable data the ohmic resistance between working electrode and reference electrode, mostly due to the resistance between the electrode surface and the connection of the reference electrode, was measured and the curves are corrected with the voltage drop induced by this resistance. Thus, the high frequency impedance between working electrode and reference electrode was measured at 20 kHz and attributed to the ohmic resistance (*R*_*ohm*_). Subsequently, the product of these resistances and the current (*I*) was used to correct the voltage, *U*_*correct*_ = *U*_*measured *_− *IR*_*ohm*_. The durability test has be conducted at a constant current of 0.5 A cm^−2^ for 5 weeks and CV is randomly recorded within a potential window between − 0.21 V and 0.15 V (vs. RHE). The double-layer capacitances (C_dl_) were measured by CV cycles under the potential window of 0.3 and 0.5 V versus RHE with various scan rates from 10 to 100 mV s^−1^ in the order to calculate electrochemical active surface area of the as-prepared samples. Tafel slope was modeled by the empirical Tafel equation: *η* = *a* + *b*log|*j*|, where *η* is the overpotential, *j* is the measured current density, *b* is the Tafel slope and *a* is the constant. For the full cell testing, the electrode No. 5 and Raney-type Ni with 4 cm^2^ active areas is used as the cathode and anode, respectively. Membrane (Dapozol), which was pre-treated with 1.0 M KOH for 24 h and then washed with Deionized water (DI) water, is placed between two coated electrodes and tested in 30 wt.% KOH at two different temperatures of 70 and 90 °C by recording polarization curves up to 2 A cm^−2^ with the slow scan rate of 10 mA s^−1^, after 30 min activation at constant current 0.2 A.

## Supplementary information

Supplementary file1
