# Supplementary material for: Improving plasma sprayed Raney-type nickel–molybdenum electrodes towards high-performance hydrogen evolution in alkaline medium
Source: Sci Rep. 2020 Jul 2;10:10948. doi: 10.1038/s41598-020-67954-y (PMC7331817; doi:10.1038/s41598-020-67954-y)
Supplement: Supplementary file 1 — Supplementary file1 [file 41598_2020_67954_MOESM1_ESM.docx]

Supplementary Information

**Improving plasma sprayed Raney-type nickel-molybdenum electrodes towards high-performance hydrogen evolution in alkaline medium**

Fatemeh Razmjooei^a^†*, Taikai Liu^ab^†, Daniela Aguiar Azevedo^ac^, Efi Hadjixenophontos^a^, Regine Reissner^a^, Günter Schiller^a^, Syed Asif Ansar^a^* & Kaspar Andreas Friedrich^ad^

*^a^ Institute of Engineering Thermodynamics, German Aerospace Center, Pfaffenwaldring 38-40, 70569 Stuttgart, Germany*

*^b^ Guangdong Institute of New Materials, Changxing Road No.363, 610565 Guangzhou, China*

*^c^ Department of Chemical Engineering, Faculdade de Engenharia, Universidade do Porto, R. Dr. Roberto Frias 291, 4200-465 Porto, Portugal*

*^d^ University of Stuttgart, Institute of Building Energetics, Thermal Engineering and Energy Storage (IGTE), Pfaffenwaldring 31, 70569 Stuttgart, Germany*


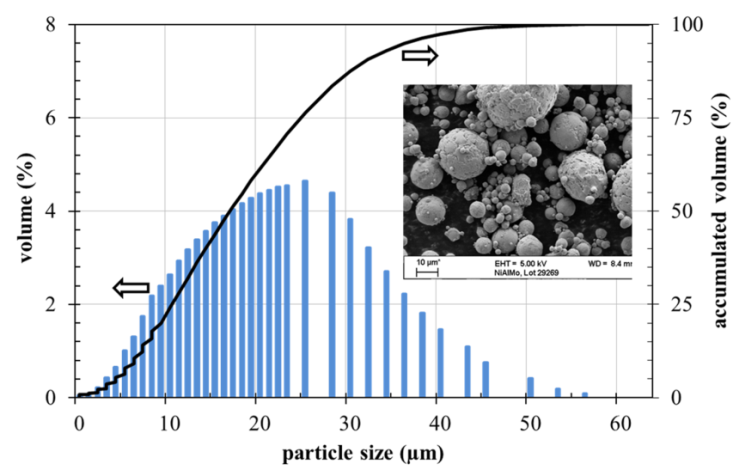


**Figure S1.** The corresponding pore size distribution curves of NiAlMo powder. Inset shows the corresponding SEM image of NiAlMo powder.


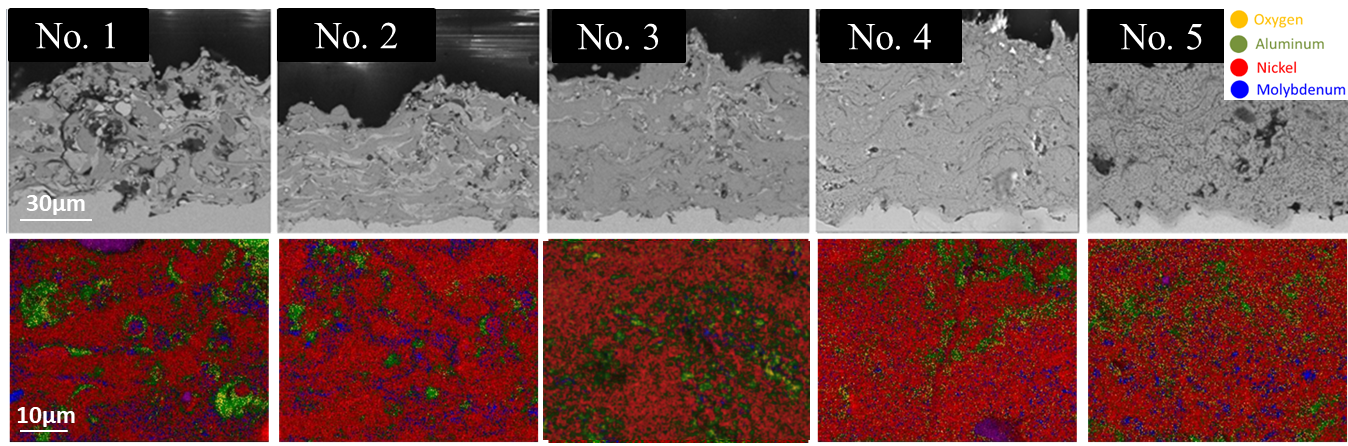


**Figure S2.** SEM images and the corresponding element mapping images of all APS-based Raney-type Ni-Mo electrodes after activation.


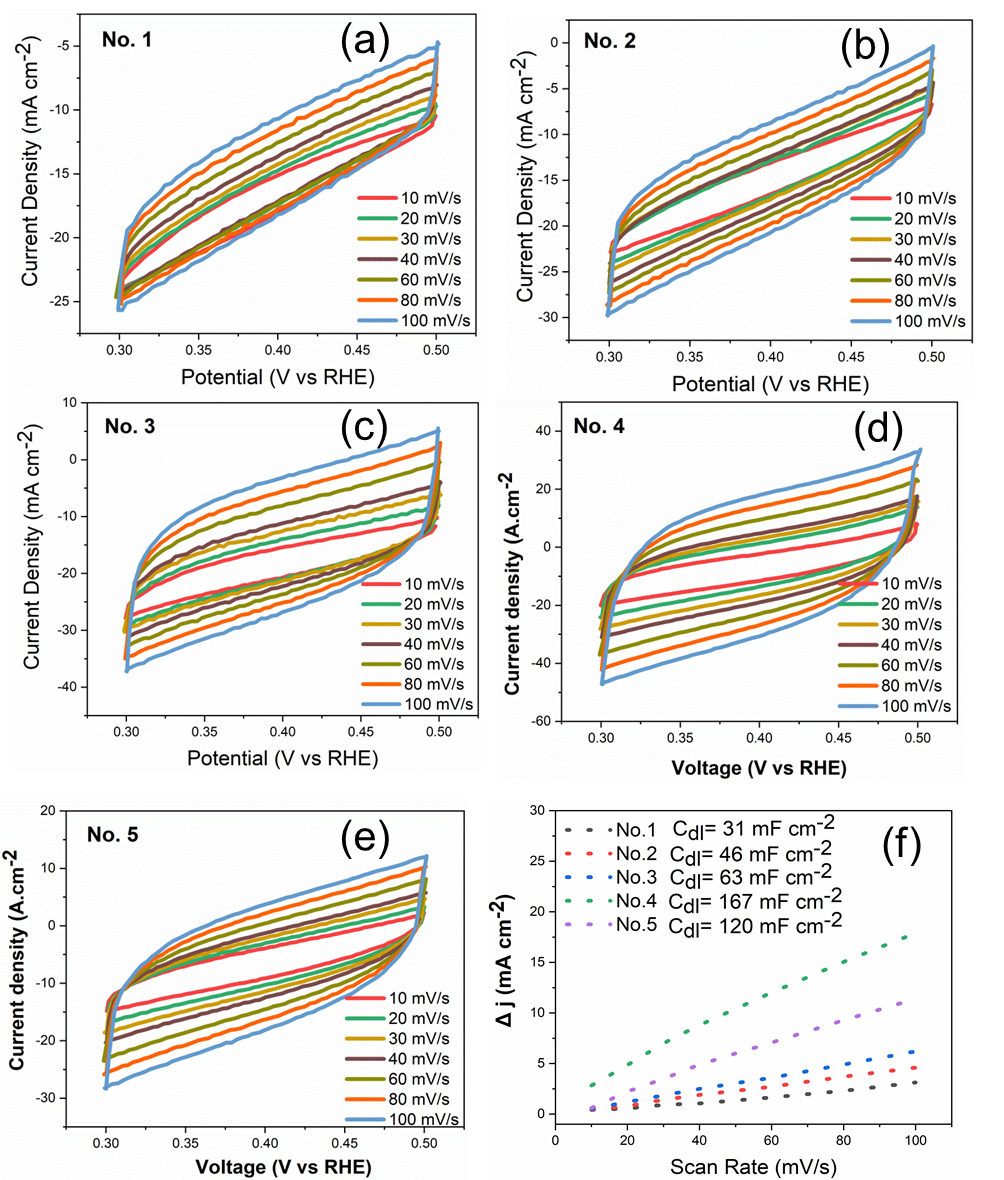


**Figure S3.** CV curves of all APS-based Raney-type Ni-Mo electrodes (a) No. 1, (b) No. 2 (c) No. 3 (d) No. 4 and No.5 in the range of 0.3 to 0.5V measured in different scan rate. (e) Corresponding current density scan rate curves to estimate the *C*_dl_ and relative electrochemically active surface area.


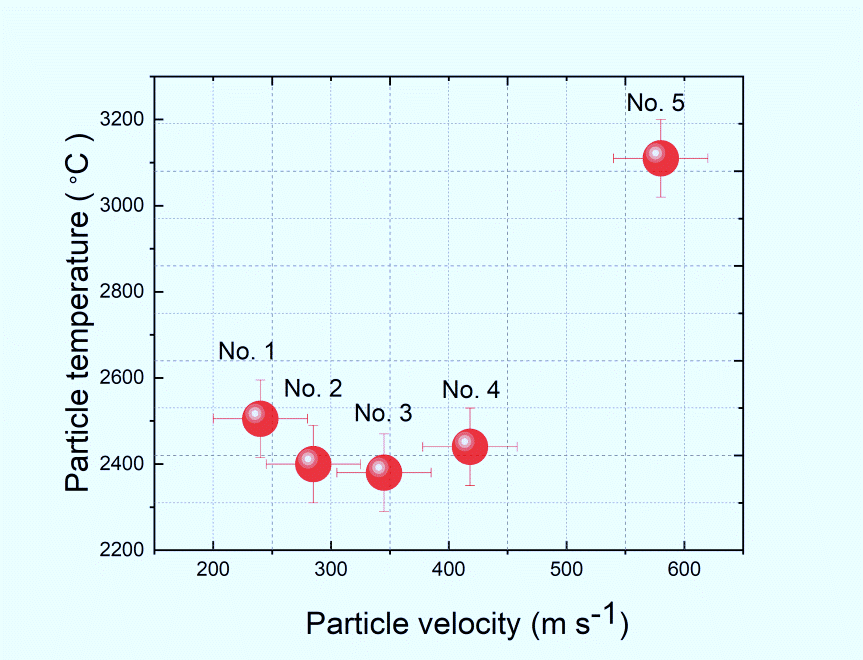


**Figure S4.** Temperature versus velocity obtained on the centerline of the plasma torch at 170 mm to the exit of the nozzle.

**Table S1.** Operating parameters of APS-based Raney-type Ni-Mo as HER electrodes.

| Samples | input gas (Ar+H_2_) | input power | spray distance | torch speed | preheating | exposing duration |
| --- | --- | --- | --- | --- | --- | --- |
|  | L.min^-1^ | kW | mm | mm.s^-1^ | °C | x10^-3^ s |
| No. 1 | 42 | 38 | 60 | 600 | 250 | 2.510 |
| No. 2 | 63 | 42 | 60 | 600 | 250 | 2.069 |
| No. 3 | 84 | 46 | 60 | 600 | 250 | 1.714 |
| No. 4 | 105 | 50 | 60 | 600 | 250 | 1.442 |
| No. 5 | 124 | 59 | 60 | 600 | 250 | 1.034 |

**Table S2.** Chemical composition of APS-based Raney -type Ni-Mo electrodes after activation by SEM/EDX.

| Sample | | Mass fraction (wt.%) | | | |
| --- | --- | --- | --- | --- | --- |
|  |  | O | Al | Ni | Mo |
| as-sprayed  (EDX) | No.1-BA | 5.94 | 28.47 | 45.98 | 16.61 |
|  | No.2-BA | 4.09 | 32.3 | 45.25 | 18.36 |
|  | No.3-BA | 3.2 | 35.58 | 43.78 | 17.44 |
|  | No.4-BA | 2.15 | 37.44 | 44.04 | 16.37 |
|  | No.5-BA | 2.01 | 37.41 | 44.36 | 16.22 |
| activated  (EDX) | No. 1 | 8.5 | 25.8 | 50.1 | 15.6 |
|  | No. 2 | 9.5 | 27.5 | 51.7 | 10.9 |
|  | No. 3 | 9.9 | 22.5 | 58.3 | 9.30 |
|  | No. 4 | 10.2 | 19.0 | 63.3 | 7.50 |
|  | No. 5 | 11.1 | 11.4 | 71.5 | 6.0 |

**Table S3.** Spot composition analysis established by EPMA for electrode No. 1, No. 2 and No. 3.

| **wt% No.** | | **P1** | **P2** | **P3** | **P4** | **P5** | **P6** |
| --- | --- | --- | --- | --- | --- | --- | --- |
| **No. 1** | O | 1.30 | 24.08 | 0.44 | 15.30 | 47.06 | Pore |
|  | Al | 25.91 | 7.69 | 22.71 | 19.03 | 52.94 |  |
|  | Ni | 49.56 | 61.67 | 53.25 | 60.39 | 0 |  |
|  | Mo | 23.23 | 6.56 | 23.60 | 5.28 | 0 |  |
| **No. 3** | O | 8.52 | 20.86 | 0.57 | 12.97 | 47.05 | Pore |
|  | Al | 3.86 | 6.93 | 21.89 | 5.15 | 52.95 |  |
|  | Ni | 60.22 | 60.62 | 58.83 | 60.25 | 0 |  |
|  | Mo | 27.40 | 11.59 | 18.72 | 21.64 | 0 |  |
| **No. 5** | O | 16.99 | 21.12 | 20.71 | 26.62 | 39.99 | Pore |
|  | Al | 15.75 | 10.80 | 10.75 | 8.46 | 45.01 |  |
|  | Ni | 42.81 | 57.83 | 57.44 | 44.26 | 4.50 |  |
|  | Mo | 24.45 | 10.25 | 11.10 | 20.67 | 10.50 |  |

**Table S4.** Summary of the HER catalytic activity of representative NiMo alloy in alkaline solutions

| Catalyst | Electrolyte | *η* (mV) | *j* (mA·cm^-2^) | Ref. |
| --- | --- | --- | --- | --- |
| No.4 | 30 wt.% KOH | 80 | 200 | This work |
| NiMo | 6.0 M KOH | 200 | 300 | 1 |
| NiMo | 6.0 M KOH | 185 | 300 | 2 |
| NiMo | 5.0 M KOH | 150 | 700 | 3 |
| NiMo | 6.0 M NaOH | 250 | 140 | 4 |
| NiMo | 6.0 M KOH | 500 | 200 | 5 |

1. Raj, I.A. & Vasu, K.I. *Journal of Applied Electrochemistry* *20*, 32-38 (1990).

2. Raj, I.A. *J Mater Sci* **28**, 4375-4382 (1993).

3. Tang, X., Xiao, L., Yang, C., Lu, J. & Zhuang, L. *International Journal of Hydrogen Energy* ***39***, 3055-3060 (2014).

4. Xia, M., Lei, T., Lv NL, *Int J Hydrogen Energy* **39**, 4794-4802, (2014).

5. Wu, L., He, Y.H., Lei, T., Nan, B., Xu, N.P., Zou, J., Huang, B.Y., CT, L. *Energy* **63**, 216-224, (2013).
